# Editorial: Generation Growbots: Materials, Mechanisms, and Biomimetic Design for Growing Robots

**DOI:** 10.3389/frobt.2021.711942

**Published:** 2021-06-15

**Authors:** Barbara Mazzolai, Ian Walker, Thomas Speck

**Affiliations:** ^1^ Bioinspired Soft Robotics Laboratory, Istituto Italiano di Tecnologia, Genova, Italy; ^2^ Department of Electrical and Computer Engineering, Clemson University, Clemson, SC, United States; ^3^ Botanic Garten and Cluster of Excellence livMatS, University of Freiburg, Freiburg, Germany

**Keywords:** growing robotics, soft robotics, bioinspired robotics, biomimetic architecture, plant biology

Plants are the dominant life form on the planet, accounting for over 80% of its biomass (Thompson, 2018). Plants are adapted to and thrive in virtually all environments, both natural and human-adapted, across the globe. In achieving this widespread presence, plants exhibit a significant range of structures and operational strategies. On the one hand, many key aspects of plant biology remain imperfectly understood, and the possibilities for plant-inspired engineering remain largely unexplored. On the other hand, increasing interest in plant-inspired research can be observed in architecture and technology in general over the last decades (cf. Speck and Speck 2019). More recently, plants have also started to represent models in robotics (Mazzolai et al., 2010; Lastinger et al., 2019; Sadeghi et al., 2020; Wooten et al., 2018), especially for the design of systems that have to deal with unstructured environments and require advanced capabilities of soft interaction, adaptation, and self-morphing. With this view, the goal of this special issue is to illustrate the potential of identifying principles from plant growth and movement suitable for engineering, and the adaptation of those principles to the new emerging field of “growing” robots, or Growbots.

The field of robotics has expanded rapidly over the past 25 years. Important advances in robotic design, planning, locomotion, and manipulation have been inspired and driven by insights gained from biology, notably in the structure and behavior of animals. However, to date very little attention has been paid by roboticists to the multitude of “existence proofs” provided by plants.

In this Research Topic, which is based on the contributions presented at the 2019 Robotics Science and Systems (RSS) workshop “Generation GrowBots” (June 22, 2019 in Freiburg, Germany), we present a research topic of nine articles focused on the intersection of robotics and plant biology. The articles are authored by a highly interdisciplinary group of domain experts, bringing together natural scientists and engineers, including experts in material science, soft robotics, plant biology, and architecture to present new scientific discoveries on plants and technological advances relevant to continuum, soft, adaptable, and growing robots. Collectively, the articles are representative of the current state of the art in the emerging area of plant-inspired robotics. Trends, frontiers and potential applications for a variety of high-tech sectors are discussed.

Under the Research Topic “Generation GrowBots” contributing authors discuss the science and technologies of the new field of plant-inspired robotics and growing robotics, exploring the materials, mechanisms and behavioral strategies as the basis of a new paradigm for robot mobility inspired by the moving-by-growing ability of plants. Plants show unique capabilities of endurance and movement by growth. Growth allows plants to strongly adapt the body morphology to different environmental conditions, and to move in search for nutrients and light or for protection from harmful agents. Because of these features, together with plant biologists and materials scientists, engineers are deeply investigating the biomechanics, materials, energy efficiency mechanisms, and behavior of a variety of plant species, to take inspiration for the design of multi-functional and adaptable technologies, and for the development of a new class of low-mass, low-volume robots endowed with new and unprecedented abilities of movement. With their capability to better challenge unstructured and extreme environments, soft, self-morphing, growing machines will have potential applications in a variety of sectors, including the exploration and monitoring of archaeological sites, unknown/challenging terrestrial or extra-terrestrial areas, as well as novel technological systems for the advancement of future urban architectures.

The topics of the nine articles in the present issue on “Generation GrowBots” vary in focus, but all address the overall theme of plant-based movement and its potential adaptation to robots. Two articles (Gallentine et al.; Geer et al.) introduce new robotic structures based on curling structures in fruit awns and climbing plants. The two examples cover a huge size range. The biomimetic robotic manipulator presented by Geer et al. is inspired by the ultrastructure of the cell wall of awns showing a helical cellulose fiber arrangement which allows for humidity driven awn movement. The concepts for transfer to motile structure in robots presented by Gallentine et al. are based on the macroscopic structure and movement of liana stems and tendrils and the finding that many climbing plants use curling and/or twining of their stems or tendrils for stiffening (braided stems) or securing attachment (tendrils). They show that these systems represent interesting models for new types of climbing plant-inspired soft robots.

The nature of movement in plants, and the consequent implications for plant-inspired robots, are considered by Frazier et al., and models of plant growth aimed at implementation in robots are presented by Porat et al.. These two contributions prove that for a successful transfer of motion principles and movements in plants to soft robots and other types of soft machines, a thorough analysis of these movements in plants using a combination of experimental and modeling approaches are a prerequisite. Without a basic and quantitative understanding of the form-structure-function relation of the plant organs used as concept generators for moving GrowBots the potential of plant-inspired approach cannot fully be used.

Realizations of vine-inspired growing robots are described in (Blumenschein et al.), with review on recent work on robots that “grow” via pressure-driven eversion, referred to as “everting vine robots,” due to a movement pattern that allows the soft systems to explore the environment. Designs based on eversion can extend over long distances (tens of meters), and offer numerous potential application novel to robotics.

Mechanical adaptations in climbing plants are considered by Soffiatti and Row, who analyzed the mechanics and underlying structure of a climbing cactus, which proves to be a suitable concept generator for shape adaptive and shape memory compound polymer materials systems which can be produced by additive manufacturing.

Root systems, a critical but generally neglected aspect of plant structures, are discussed in (Stachew et al.) as they offer promising strategies for the design of civil and coastal infrastructure, such as adaptivity, multi-functionality, self-healing, mechanical and chemical soil attachment. Using a biomimetic methodology, the work presents the potential of root-inspired designs for building foundations and coastal infrastructures that prevent soil erosion, anchor structures, penetrate soils, and provide natural habitat.

The research review by Esser et al. summarizes the current state of the art in constructing Artificial Venus Flytraps (AVTs), which represent iconic examples of plant-inspired soft machines. The article gives an outlook on the work done on ATVs in the Cluster of Excellence *liv*MatS. AVTs are prime examples for shifting/blurring the boundaries between living and life-like but entirely technical systems (Speck and Speck, 2021). However, in addition to examining the question of dissolving this boundary the article offers some interesting potential applications for plant-inspired soft robots and building hulls in architecture.

Last but not least, as it has been 10 years since the publication of the first article looking at plants as a biomechatronic system and creating a bidirectional link between robotics and plant biology, the mini-review by Mazzolai et al. offers a brief overview of the fundamental aspects related to a bioengineering approach in plant-inspired robotics. The article analyses the works in which both biological and engineering aspects have been investigated, and highlights the key elements of plants that have been milestones in the pioneering field of growing robots.

We hope that the special issue will prove informational and inspirational to readers new to the topic, and also be a valuable resource for current and future researchers in the area.
